# Early-Life Antibiotic Cocktail Intervention Alters Cecal Microbiota Composition and Metabolic Profiles in Suckling Rats

**DOI:** 10.3390/microorganisms14092048

**Published:** 2026-09-14

**Authors:** Sen Chen, Shiyi Tian, Zhihan Dong, Xueyao Zheng, Huanhuan Shan, Tian Huan, Yuyou Duan, Jue Wang

**Affiliations:** 1 Laboratory of Stem Cells and Translational Medicine, Center for Medical Research on Innovation and Translation, Institute of Clinical Medicine, The Second Affiliated Hospital, School of Medicine, South China University of Technology, Guangzhou 510006, China; 2 College of Animal Science and Technology, Jiangxi Agricultural University, Nanchang 330045, China; 3 Laboratory of Stem Cells and Translational Medicine, Institute for Life Science, School of Medicine, South China University of Technology, Guangzhou 510006, China

**Keywords:** suckling rat pups, antibiotic-perturbed dysbiosis model, antibiotic intervention, gut microbiota, metabolomics

## Abstract

The early-life lactational window is critical for gut microbial colonization, intestinal maturation, and long-term host metabolic and immune programming. Although antibiotic-induced microbiota perturbation models are widely applied to investigate microbiota function, the overall microbiota perturbation efficiency and comprehensive metabolic alterations induced by broad-spectrum antibiotics during the lactational stage remain poorly characterized. In this study, neonatal SD rat pups were continuously administered a broad-spectrum antibiotic cocktail to establish a lactational antibiotic-perturbed dysbiosis model. The early-life antibiotic intervention induced mild impairment of the intestinal structure and significantly increased the relative hindgut weight. 16S rRNA sequencing demonstrated significant reductions in microbial α-diversity, profound remodeling of gut microbial communities, extensive depletion of core beneficial genera including *Akkermansia*, *Bifidobacterium*, and *Lactobacillus*, and significant enrichment of opportunistic pathogens. Untargeted metabolomics further revealed widespread cecal metabolic perturbations following antibiotic-induced microbiota perturbation, including decreased biosynthesis of microbiota-derived vitamins, and comprehensive disorders in lipid, tyrosine, pteridine, and steroid hormone metabolism. Collectively, this study systematically characterized the microbiota perturbation efficiency and multiomic metabolic phenotypes of antibiotic-induced microbiota perturbation in suckling rats, clarifying the regulatory effects of lactational microbial perturbation on intestinal microecology and host metabolic homeostasis. These findings provide fundamental phenotypic data for the application of early-life antibiotic-perturbed dysbiosis models and further mechanistic exploration of host–microbe crosstalk during the critical developmental window.

## 1. Introduction

Early-life mucosal microbial colonization constitutes a critical developmental window for the maturation and immune education of the mammalian host immune system [1,2]. Microbial homeostasis during this period persistently shapes host immune tolerance and exerts long-term, even irreversible, programming effects on the susceptibility to chronic diseases in later life [3,4]. The disruption of early-life microbiota homeostasis impairs the dynamic crosstalk between the host and commensal microbes, hinders the developmental priming of immune cells, and substantially elevates the risk of immune-mediated disorders, including inflammatory bowel disease, allergies, and asthma [5,6]. A recent study further demonstrated that depletion of short-chain fatty acid-producing bacteria in early life remodels gene expression and H3K27 histone acetylation modifications in hematopoietic stem and progenitor cells, thereby establishing a persistent, transplantable atopic immune phenotype. Such microbial dysbiosis ultimately drives elevated IgE concentrations, heightened susceptibility to allergic inflammation, and compromised hematopoietic function [7]. Given the central regulatory role of the neonatal gut microbiota, deciphering causal links between microbial colonization and host health represents a core research priority in gut microecology. Functional validation approaches, including mono-bacterial colonization, strain-specific intervention, and fecal microbiota transplantation, allow the precise characterization of the discrete physiological functions of individual gut commensals [8,9]. Notably, all these functional assays are heavily dependent on microbiota-deficient animal models, underscoring the indispensable value of germ-free rodents and antibiotic-induced microbiota-perturbation models for dissecting early-life host–microbe interaction mechanisms [10].

Germ-free animals are regarded as the gold standard for investigating gut microbial functions; nevertheless, their broad application is greatly limited by high breeding costs, demanding rearing requirements, and intrinsically underdeveloped immune systems [11,12]. Accordingly, antibiotic-induced microbiota-perturbation models have become cost-effective, practical alternatives for simulating microbiota depletion and replacing germ-free animals in microbiota function research [13]. Conventional antibiotic regimens are typically delivered via drinking water, an approach only suitable for weaned animals with independent drinking capacity [11,13]. In contrast, suckling rat pups obtain nutrition exclusively from maternal milk and cannot ingest drugs through drinking water, rendering traditional water-supplemented antibiotic interventions infeasible for lactational neonatal animal models. Several recent studies have generated antibiotic-perturbed dysbiosis models in suckling rodents using oral-bolus antibiotic administration and confirmed that early-life microbial depletion profoundly modulates intestinal immune development [13]. Huang et al. reported that accumulation of 12R-hydroxyeicosatetraenoic acid induced by gut microbiota depletion binds the nuclear receptor Nur77, upregulates T-bet expression in T lymphocytes, facilitates the differentiation of NKp46+ group 3 innate lymphoid cells, and boosts IFN-γ secretion to maintain intestinal innate immune homeostasis [14]. Although previous work has verified that neonatal antibiotic exposure reshapes host immune phenotypes, systematic quantitative assessment of indigenous gut microbiota clearance efficiency achieved by broad-spectrum antibiotic cocktails in suckling rat pups is still lacking [14,15]. Furthermore, the comprehensive remodeling of cecal metabolomic composition and metabolic signatures triggered by lactational microbiota depletion remains incompletely characterized.

In the present study, we established an early-life antibiotic-perturbed dysbiosis model via continuous oral-bolus delivery of a broad-spectrum antibiotic cocktail to neonatal SD rat pups. We systematically explored the impacts of early-life ABX intervention on intestinal morphology, organ development, gut microbiota community assembly, and cecal metabolomic signatures in suckling rats. This study aimed to elucidate how early-life microbiota perturbation regulates intestinal microecological homeostasis and host metabolic phenotypes, provide baseline phenotypic data regarding microbial clearance features and metabolic profiles in lactational antibiotic-perturbed dysbiosis pup models, and offer methodological references for probing early-life host–microbe crosstalk.

## 2. Materials and Methods

### 2.1. Animal Experimental Design and Sampling

Neonatal Sprague-Dawley (SD) rat pups were used in this study. All experimental animals were purchased from Hunan SJA Laboratory Animal Co., Ltd. (Changsha, China). Maternal dams were housed under standardized laboratory conditions: ambient temperature of 22–25 °C, relative humidity maintained at 50% ± 5%, and a 12 h light/dark cycle with free access to standard rodent chow and tap water. In total, neonatal rat pups were derived from four independent pregnant dams, with litter sizes of 16, 15, 13 and 12 pups. To minimize confounding effects originating from maternal genetics and milk composition, pups within each litter were evenly split into the control group (CON) or broad-spectrum antibiotic cocktail group (ABX). The individual suckling pup was defined as the experimental unit. Eight biological replicates for both control and ABX groups were obtained from four independent litters, with two pups sampled per group from each litter. From postnatal day (PND) 0 to PND 14, all rat pups received twice-daily ABX intervention at 08:00 and 20:00 (corresponding to light-on and light-off of the animal facility). Manipulation was postponed if dams were nursing until nursing behavior ceased. Antibiotic solution delivery was performed using sterile Eppendorf pipette tips utilizing the natural suckling reflex of neonatal pups. The pipette tip was placed only inside the oral cavity without advancing into the esophagus; liquid solution was administered drop-wise to the oral vestibule for spontaneous swallowing by the pups. The administration volume was adjusted weekly according to pup body weight, ranging from 10 μL in the first week to 25 μL per dose in the second postnatal week. The CON group received an equal volume of sterile PBS, while the ABX group received an antibiotic cocktail dissolved in sterile PBS, containing final working concentrations of 3.75 mg/mL ampicillin, 3 mg/mL vancomycin and 6 mg/mL neomycin. On PND 14, all suckling pups were humanely euthanized by cervical dislocation under aseptic conditions. Ileal tissues were isolated for hematoxylin–eosin (H&E) histological staining. The liver, foregut and hindgut were dissected and weighed to calculate relative organ weight, defined as (organ wet weight/whole body weight) × 100%. Cecal digesta were immediately collected, snap-frozen in liquid nitrogen, and stored at −80 °C for subsequent 16S rRNA amplicon sequencing and untargeted metabolomic analysis.

### 2.2. Microbial DNA Extraction, 16S rRNA Gene Amplification and High-Throughput Sequencing

Total genomic DNA of gut-derived microorganisms was isolated from cecal digesta samples with a FastPure Stool DNA Isolation Kit (MJYH, Shanghai, China), strictly following the manufacturer-recommended operational procedures [16]. Quality and yield of recovered DNA were assessed by agarose gel electrophoresis together with spectrophotometric measurements. The V3–V4 hypervariable segment of the bacterial 16S rRNA gene was amplified with the universal primer set as described in a previous study [16]. Target fragments were amplified through polymerase chain reaction with consistent thermal cycling parameters. Resulting PCR amplicons were resolved on 2% agarose gels, subsequently purified utilizing an AxyPrep DNA Gel Extraction Kit (Axygen Biosciences, Union City, NJ, USA), and subjected to precise concentration determination. Purified PCR products were mixed at equal molar concentrations before paired-end high-throughput sequencing on an Illumina Nextseq2000 instrument (Illumina, San Diego, CA, USA). Sequencing services were completed by Majorbio BioPharm Technology Co., Ltd. (Shanghai, China). Raw sequencing reads were preserved for subsequent bioinformatics workflows.

### 2.3. Sequence Processing and Bioinformatic Analysis

Raw FASTQ sequencing reads were subjected to quality filtering, trimming and assembly steps to generate clean sequence tags. Clean reads were binned into operational taxonomic units (OTUs) at a nucleotide similarity cutoff of 97%, and the most abundant sequence within each OTU was selected as the representative taxonomic read. Subsequent comprehensive bioinformatic profiling of cecal microbiota was performed on the Majorbio Cloud Platform (https://cloud.majorbio.com; accessed on 12 October 2022) [16]. Microbial α-diversity metrics were calculated using MOTHUR v.1.30.1 to evaluate community richness and species evenness. Principal coordinate analysis (PCoA) based on Bray–Curtis dissimilarity metrics was implemented via the R package Vegan v2.5-3 to characterize β-diversity and overall community separation across experimental groups. The linear discriminant analysis effect size (LEfSe) algorithm (http://huttenhower.sph.harvard.edu/LEfSe; accessed on 12 October 2022) was applied to screen discriminatory microbial biomarkers, with preset thresholds of LDA score > 2 and *p* < 0.05.

### 2.4. Sample Preparation for LC-MS/MS Untargeted Metabolomics

Consistent weight cecal digesta specimens were transferred into 2 mL grinding centrifuge tubes containing ceramic homogenization beads for metabolite extraction following standard untargeted metabolomic workflows. Briefly, pre-chilled extraction solvent spiked with the internal standard L-2-chlorophenylalanine was added to each sample. The mixtures were thoroughly homogenized with a cryogenic tissue grinder and further processed through low-temperature sonication. After 30 min incubation at −20 °C, samples were centrifuged at 13,000× *g* for 15 min under 4 °C. The resulting supernatants were transferred to sample injection vials for subsequent UHPLC-Q-Orbitrap MS/MS acquisition.

### 2.5. LC-MS/MS Metabolic Profiling

Untargeted metabolomic profiling was carried out on a Thermo Scientific UHPLC-QExactive-HFX high-resolution mass spectrometer, with sequencing services provided by Majorbio BioPharm Technology Co., Ltd. (Shanghai, China). A 3 μL aliquot of supernatant was loaded and separated on an HSST3 chromatographic column (100 mm × 2.1 mm i.d., 1.8 μm particle size). The mobile-phase gradient consisted of phase A (95% ultrapure water plus 5% acetonitrile containing 0.1% formic acid) and phase B (47.5% acetonitrile + 47.5% isopropanol + 5% ultrapure water supplemented with 0.1% formic acid). The flow rate was maintained at 0.40 mL/min, and the column oven temperature was set to 40 °C. Mass spectrometric acquisition was performed under both positive and negative electrospray ionization modes, with full scan *m/z* ranging from 70 to 1050. The sheath gas flow was set to 50 psi, auxiliary gas flow to 13 psi, and auxiliary gas temperature to 425 °C. Ion spray voltage was adjusted to 3500 V for positive-ion mode and −3500 V for negative-ion mode, while the ion transfer tube temperature was stabilized at 325 °C. A normalized collision-energy gradient (20–40–60 V) was applied for secondary fragmentation. The primary MS resolution was set to 60 000, and secondary fragment resolution was 7500.

### 2.6. Metabolite Identification and Functional Annotation

Raw LC-MS/MS spectral datasets were imported into Progenesis QI software V2.0 (Waters Corporation, Milford, MA, USA) to perform baseline correction, peak detection, peak integration, retention time alignment and peak matching, generating a standardized dataset matrix storing retention time, mass-to-charge ratio and peak intensity values for all detectable metabolites. Metabolite annotation was accomplished by matching primary and secondary MS/MS spectra against HMDB (http://www.hmdb.ca/ accessed on 12 October 2022), METLIN (https://metlin.scripps.edu/: accessed on 12 October 2022) and the in-house metabolite spectral library constructed by Majorbio. Data pretreatment and multivariate statistical computation were completed on the Majorbio Cloud Platform. The R package ropls (v1.6.2) was utilized for partial least-squares discriminant analysis (PLS-DA). Seven-fold cross-validation was adopted to evaluate model performance and mitigate overfitting risks. Differential metabolites were screened according to unified criteria: Student’s *t*-test raw *p* < 0.05, fold change > 2 or <0.5, and variable importance in projection (VIP) > 1.

### 2.7. Statistical Analysis

All quantitative experimental results are presented as mean ± standard deviation (SD). Statistical computations were implemented using SPSS 20.0 software. Between-group comparisons of phenotypic indicators were performed with independent-sample Student’s *t*-test. The individual suckling pup was defined as the experimental unit, with eight biological replicates for both control and ABX groups. A priori sample size power calculation was completed via G*Power 3.1 prior to animal experiments, assuming a medium-to-large effect size (d = 0.60), α = 0.05 and target power of 0.80. Benjamini–Hochberg false discovery rate (FDR) correction was applied to adjust *p*-values for multiple comparisons for high-dimensional datasets including 16S rRNA microbial profiling and untargeted metabolomics. Adjusted q-values below 0.05 were regarded as statistically significant for omics-derived features. For multivariate PLS-DA modeling, permutation testing was utilized to assess model robustness and reduce overfitting bias. Differential metabolites were screened combining VIP values derived from the PLS-DA model and statistical outputs from Student’s *t*-test. For phenotypic parameters without FDR adjustment, raw *p* < 0.05 was considered the threshold for statistical significance.

## 3. Results

### 3.1. Early-Life ABX Intervention Alters ILEAL Histomorphology and Visceral Organ Relative Weight in Suckling Rats

**Figure 1:**
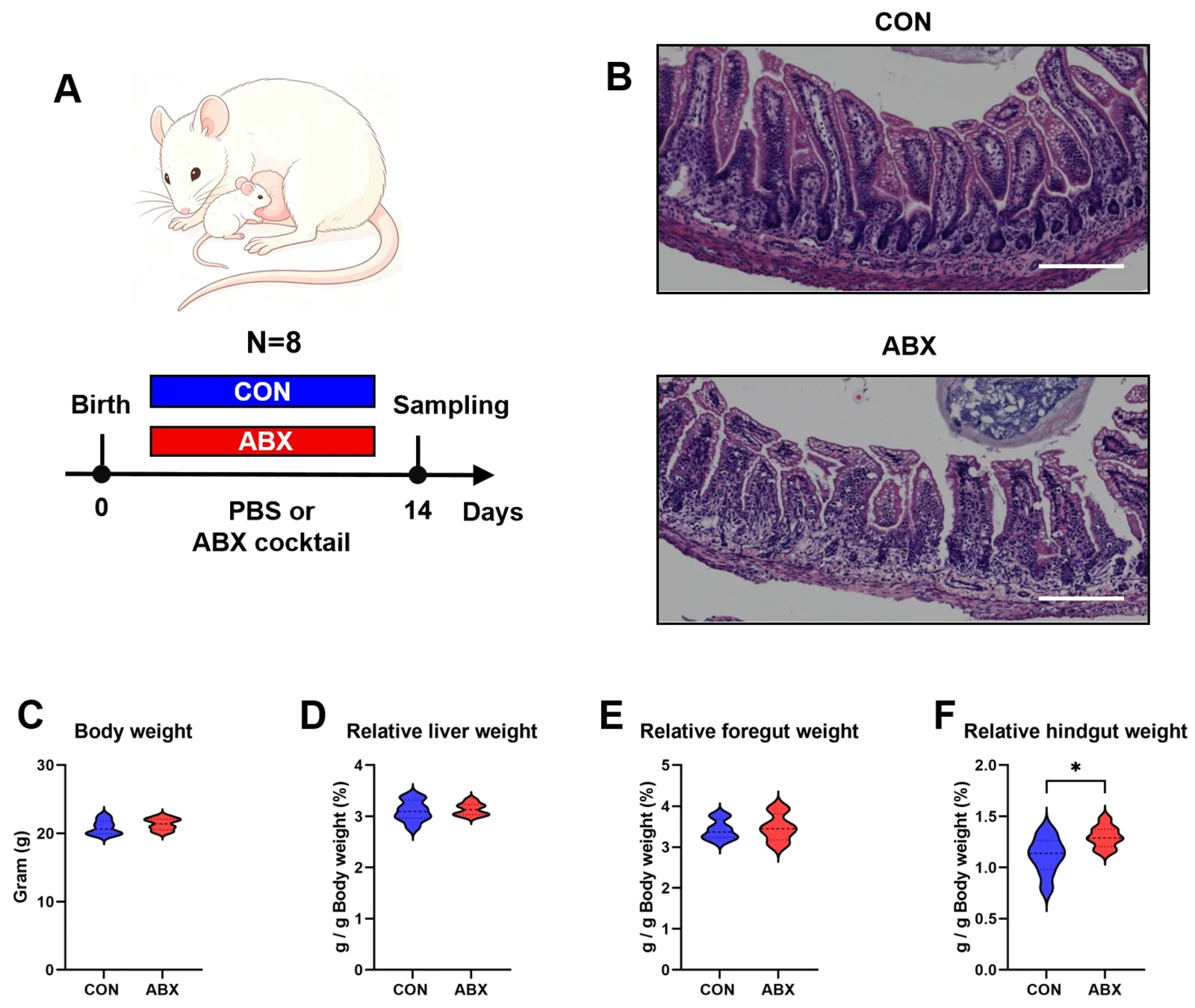
Early-life antibiotic cocktail intervention induces increased relative hindgut weight in nursing rat pups (n = 8). (**A**) Schematic diagram of animal experimental design. Nursing rat pups were treated with PBS (CON) or antibiotic cocktail (ABX) by oral bolus delivery from birth to postnatal day 14. (**B**) Representative hematoxylin–eosin staining of ileal tissues from CON and ABX groups. Scale bar: 200 μm. (**C**–**F**) Violin plots showing body weight (**C**), relative liver weight (**D**), relative foregut weight (**E**), and relative hindgut weight (**F**). Data are presented as violin plots showing distribution of individual values. * *p* < 0.05 versus CON group.

To characterize the phenotypic impacts of neonatal microbiota perturbation, suckling rat pups received daily oral bolus delivery of either a broad-spectrum antibiotic cocktail (ABX group) or an equal volume of sterile PBS (CON group) from PND 0 to PND 14 (Figure 1A). Representative H&E-stained ileal histological sections revealed mild structural impairment in the ABX group, characterized by blunted villus tips and widened subepithelial interstitial spaces, while overall villus crypt structural integrity remained intact (Figure 1B). At PND 14, whole body weight showed no statistically significant difference between the CON and ABX groups (Figure 1C). Similarly, relative liver weight and relative foregut weight were comparable between the two experimental cohorts (Figure 1D,E). In contrast, relative hindgut weight was markedly higher in antibiotic-treated suckling rats relative to control littermates (Figure 1F; *p* < 0.05). Elevated relative hindgut weight represents a canonical adaptive phenotypic signature of gut microbiota deficiency, reflecting compensatory physiological remodeling of the large intestine induced by antibiotic-mediated microbial dysbiosis.

### 3.2. Effects of Early-Life ABX Intervention on Microbial Diversity of Cecal Contents in Suckling Rats

**Figure 2:**
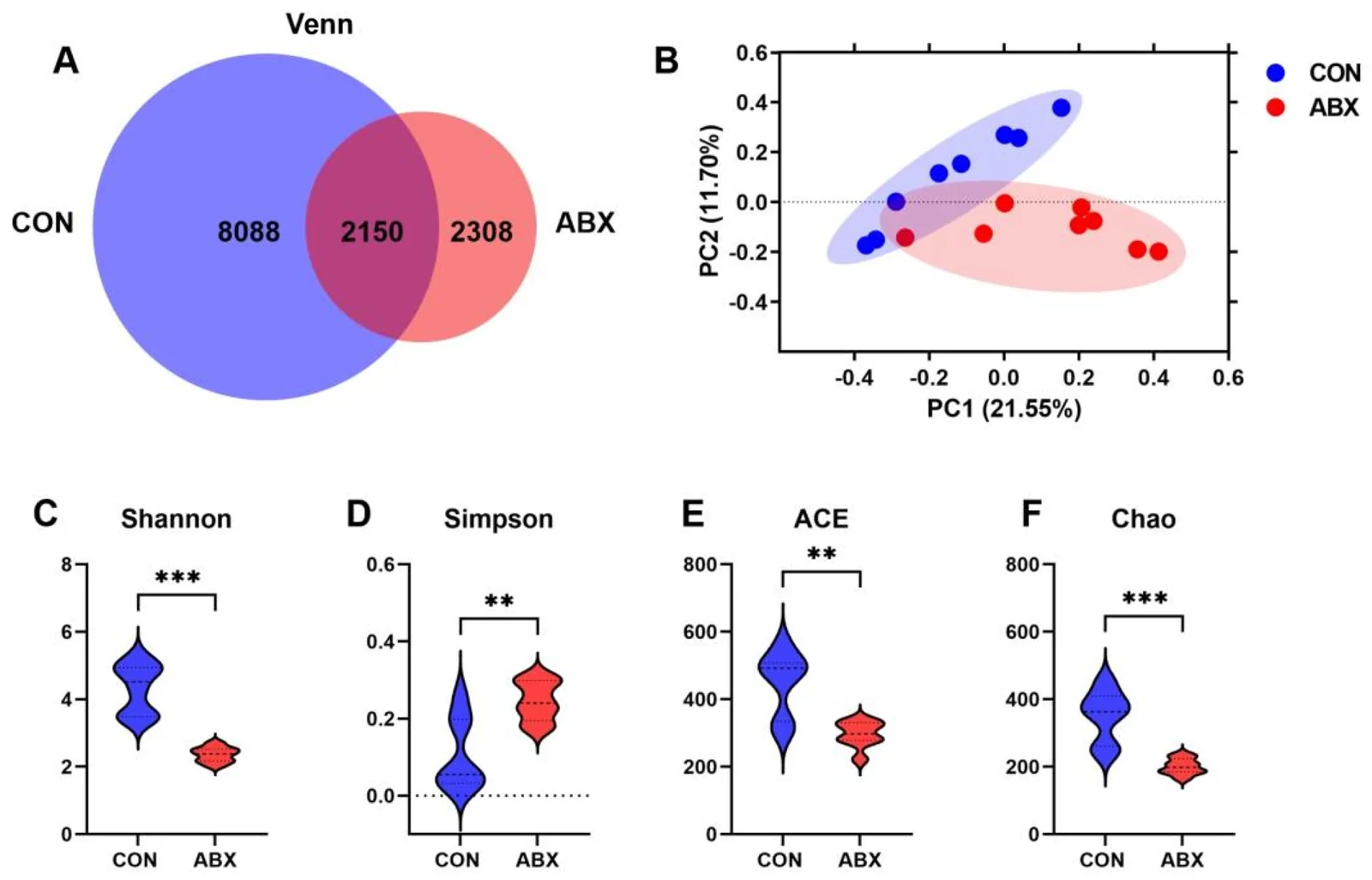
Early-life ABX intervention alters microbial diversity of cecal contents in suckling rats (n = 8). (**A**) Venn diagram showing number of unique and shared OTUs between CON and ABX groups. (**B**) PCoA plot based on Bray–Curtis distance illustrating β-diversity of gut microbiota. PC1 and PC2 explain 21.55% and 11.70% of total variance, respectively. (**C**–**F**) Violin plots of α-diversity indices: Shannon index (**C**), Simpson index (**D**), ACE index (**E**), Chao index (**F**). ** *p* < 0.01, *** *p* < 0.001 versus CON group.

Venn diagram analysis quantified shared and unique OTUs across experimental groups (Figure 2A). A total of 8088 unique OTUs were identified in the CON group, 2308 unique OTUs in the ABX group, with only 2150 OTUs shared between both cohorts. Antibiotic intervention drastically reduced the number of group-specific intestinal microbial OTUs in suckling pups. PCoA based on Bray–Curtis distance metrics was completed to assess intergroup β-diversity (Figure 2B). Samples from CON and ABX groups formed fully segregated clusters, demonstrating profound divergence in overall gut bacterial community architecture following antibiotic treatment. Subsequent α-diversity index calculation quantified community richness and evenness (Figure 2C–F). The Shannon index (*p* < 0.001) was significantly lower, while the Simpson index (*p* < 0.01) was higher in the ABX group compared with controls. Richness estimators ACE and Chao1 were markedly lower in antibiotic-exposed pups (*p* < 0.01). Collectively, these results confirm that continuous neonatal intervention with a broad-spectrum antibiotic cocktail significantly reduces gut microbial α-diversity and profoundly remodels the overall bacterial community composition of suckling rats.

### 3.3. Effects of Early-Life ABX Intervention on Microbial Composition of Cecal Contents in Suckling Rats

**Figure 3:**
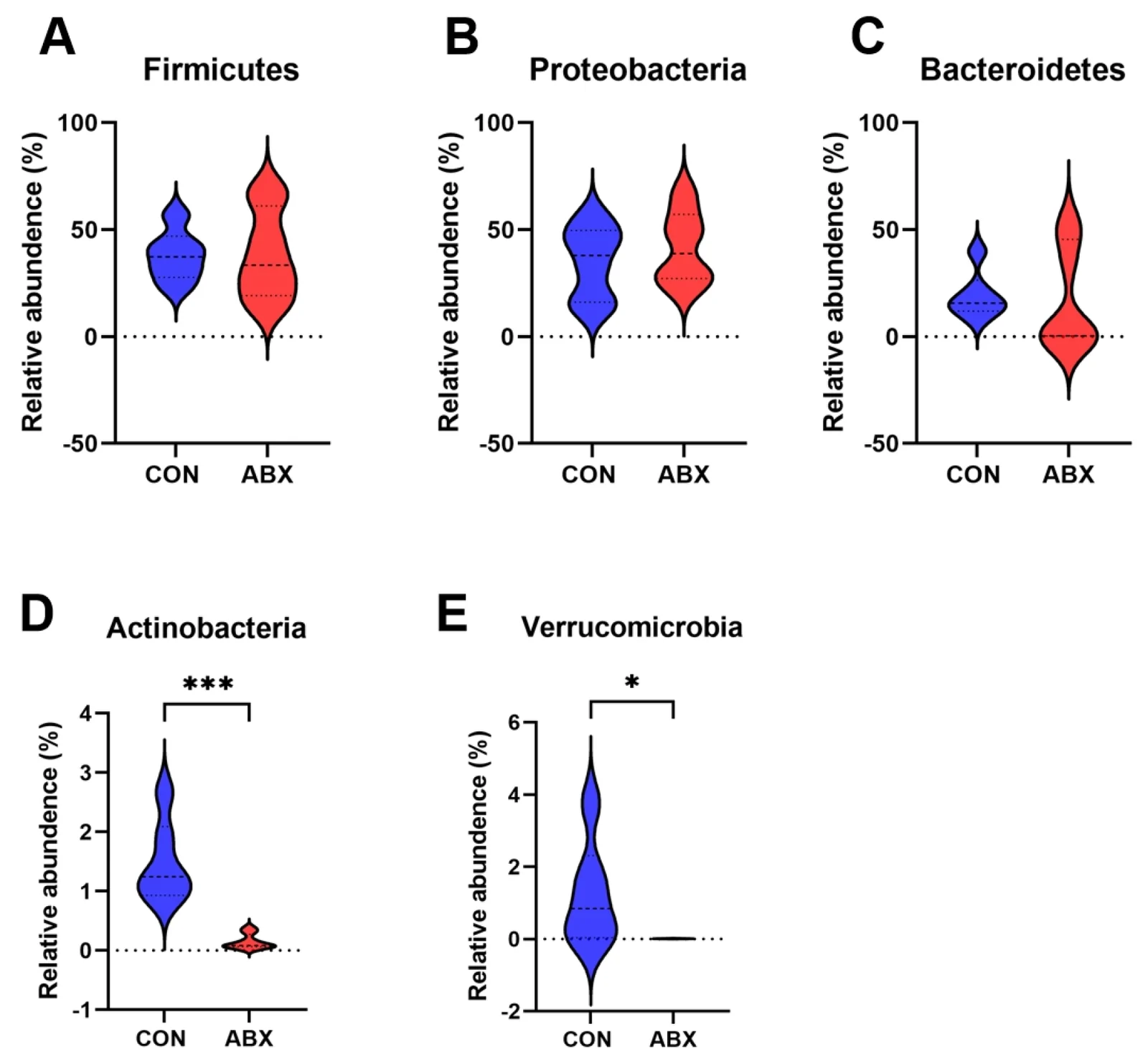
Phylum-level alterations of cecal gut microbiota during ABX intervention (n = 8). (**A**–**E**) Violin plots of relative abundance for Firmicutes (**A**), Proteobacteria (**B**), Bacteroidetes (**C**), Actinobacteria (**D**), Verrucomicrobia (**E**). * *p* < 0.05, *** *p* < 0.001 versus CON group.

Taxonomic classification identified ten bacterial phyla, among which Firmicutes, Proteobacteria and Bacteroidetes were predominant in rat cecal contents, collectively accounting for over 90% of relative abundance (Figure S1). Statistical analyses of these phyla revealed no significant differences in relative abundances of Firmicutes, Proteobacteria and Bacteroidetes between the two groups (Figure 3A–C; *p* > 0.05). Compared with the CON group, the relative abundance of Actinobacteria was significantly lower in the ABX group (Figure 3D; *p* < 0.001). Likewise, Verrucomicrobia was significantly lower in relative abundance after antibiotic treatment (Figure 3E; *p* < 0.05).

**Figure 4:**
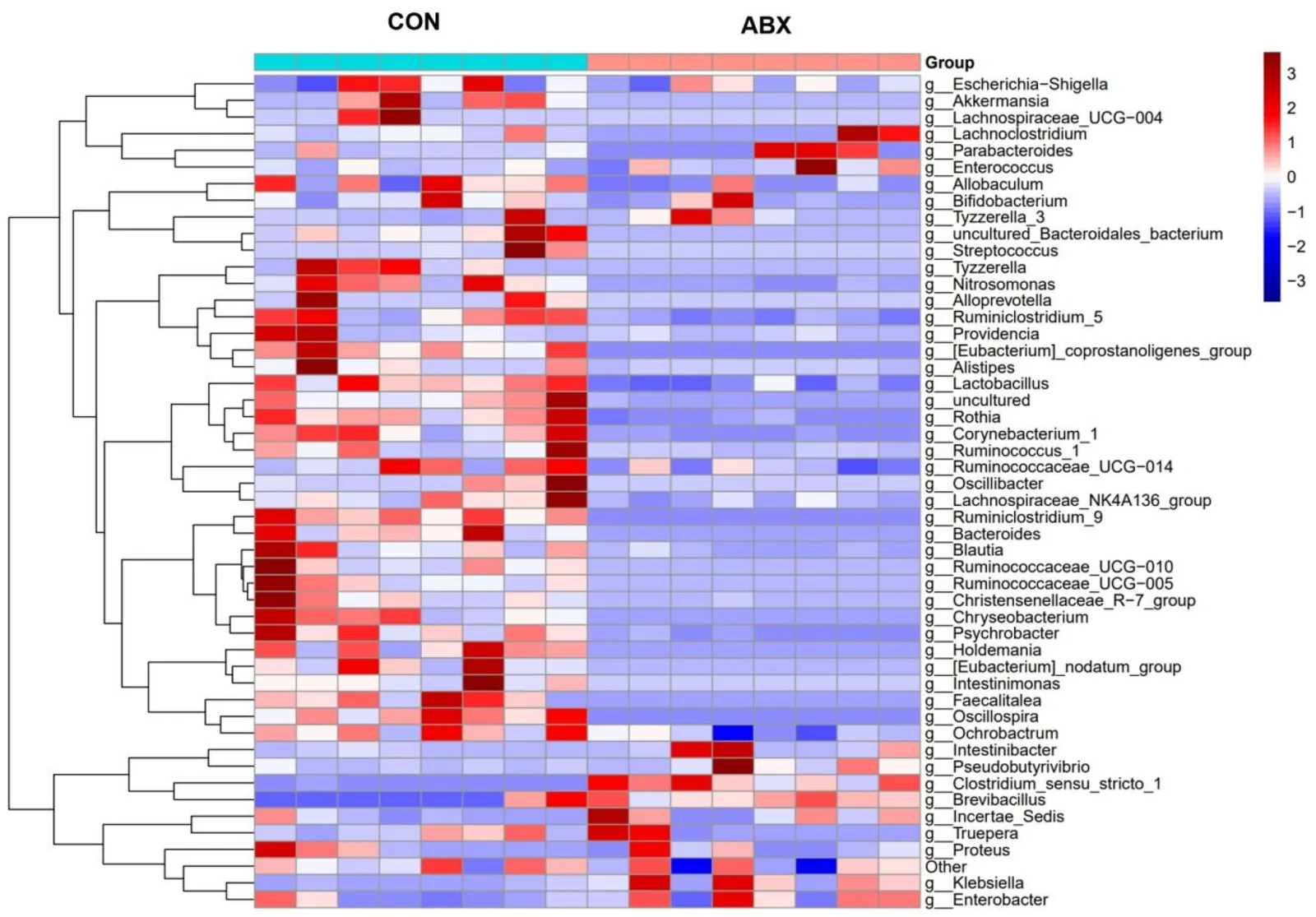
Heatmap of dominant bacterial genera in cecal microbiota (n = 8). Rows correspond to bacterial genera; columns represent individual samples. Color gradient reflects normalized relative abundance: red for high abundance and blue for low abundance. Sample groups are annotated in top bar.

**Figure 5:**
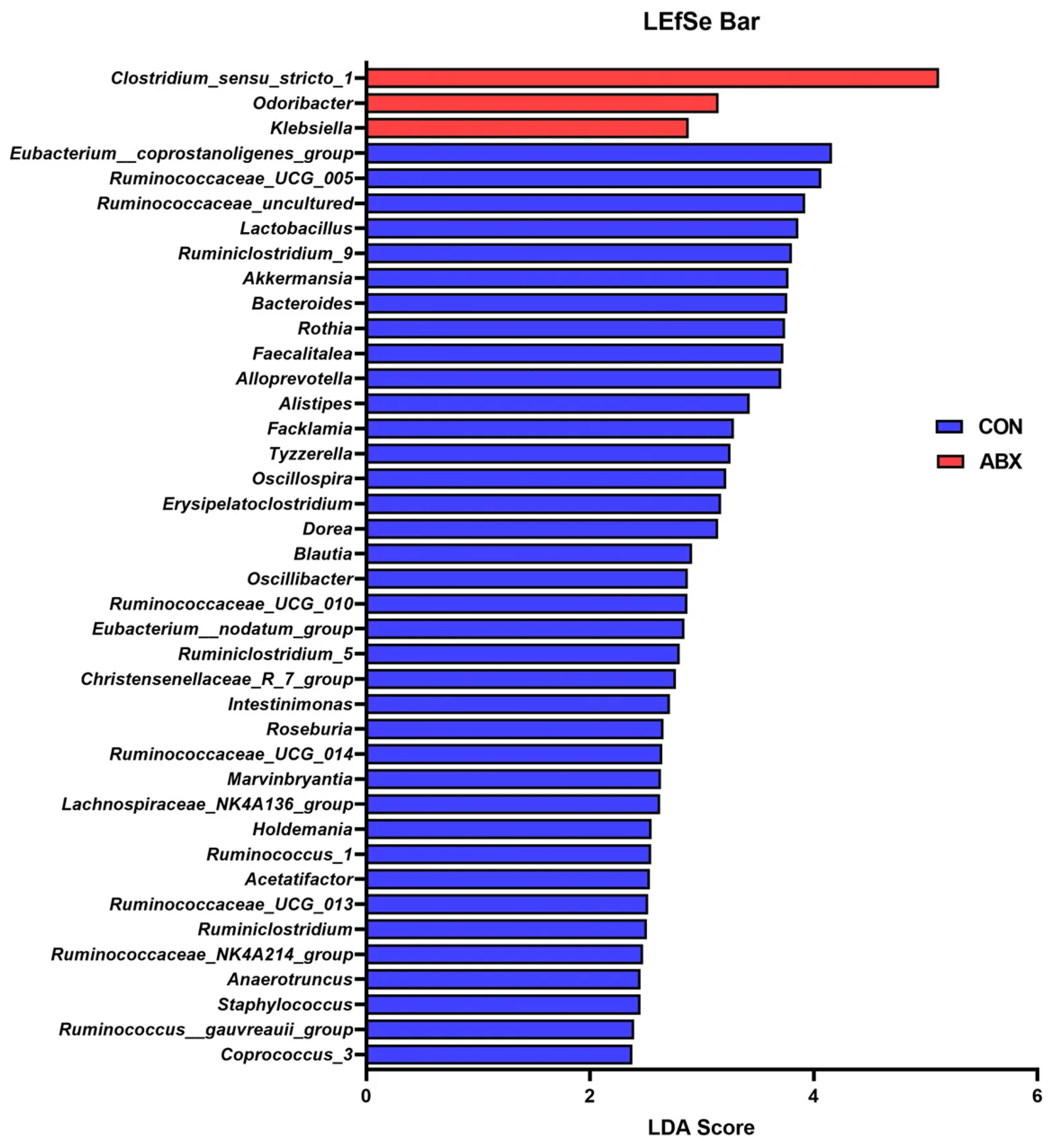
LEfSe bar plot showing differential genera between CON and ABX groups (n = 8). LDA score represents effect size of each taxon. Red bars: genera enriched in ABX group; blue bars: genera enriched in CON group.

A genus-level heatmap was constructed to visualize the abundance patterns of the predominant bacterial genera in the CON and ABX groups (Figure 4). Red indicates high relative abundance, whereas blue represents low relative abundance. Distinct genus-level microbial profiles were observed between the two groups. Most commensal genera, including *Akkermansia*, *Bifidobacterium*, *Lactobacillus*, *Alloprevotella* and multiple members of the Ruminococcaceae family, were high in abundance in the CON group and were largely depleted in the ABX group. In contrast, several genera such as *Clostridium_sensu_stricto_1*, *Klebsiella* and *Escherichia-Shigella* exhibited higher abundance in the ABX group. These results suggest that ABX intervention drastically disturbed the genus-level composition of the cecal microbiota, resulting in the depletion of beneficial commensals and enrichment of potential pathogenic genera. LEfSe analysis was performed to screen biomarker taxa between the CON and ABX groups, with the LDA score set as the threshold for effect-size evaluation (Figure 5). Red bars represent taxa enriched in the ABX group, whereas blue bars indicate taxa enriched in the CON group. Three genera, *Clostridium_sensu_stricto_1*, *Odoribacter* and *Klebsiella*, were significantly enriched in the ABX group. Among them, *Clostridium_sensu_stricto_1* showed the highest LDA score, indicating the strongest biomarker effect for antibiotic-treated animals. A large set of commensal genera were identified as signature taxa of the CON group, including *Lactobacillus*, *Akkermansia*, *Bacteroides*, *Alloprevotella*, multiple taxa belonging to the Ruminococcaceae and Lachnospiraceae families. These taxa exhibited high LDA scores, demonstrating their discriminative power for normal gut microbiota. These findings indicate that the antibiotic intervention induced the enrichment of potential pathobionts, whereas many beneficial anaerobic commensals were depleted.

### 3.4. Effects of Early-Life ABX Intervention on Cecal Metabolome Profile in Suckling Rats

**Figure 6:**
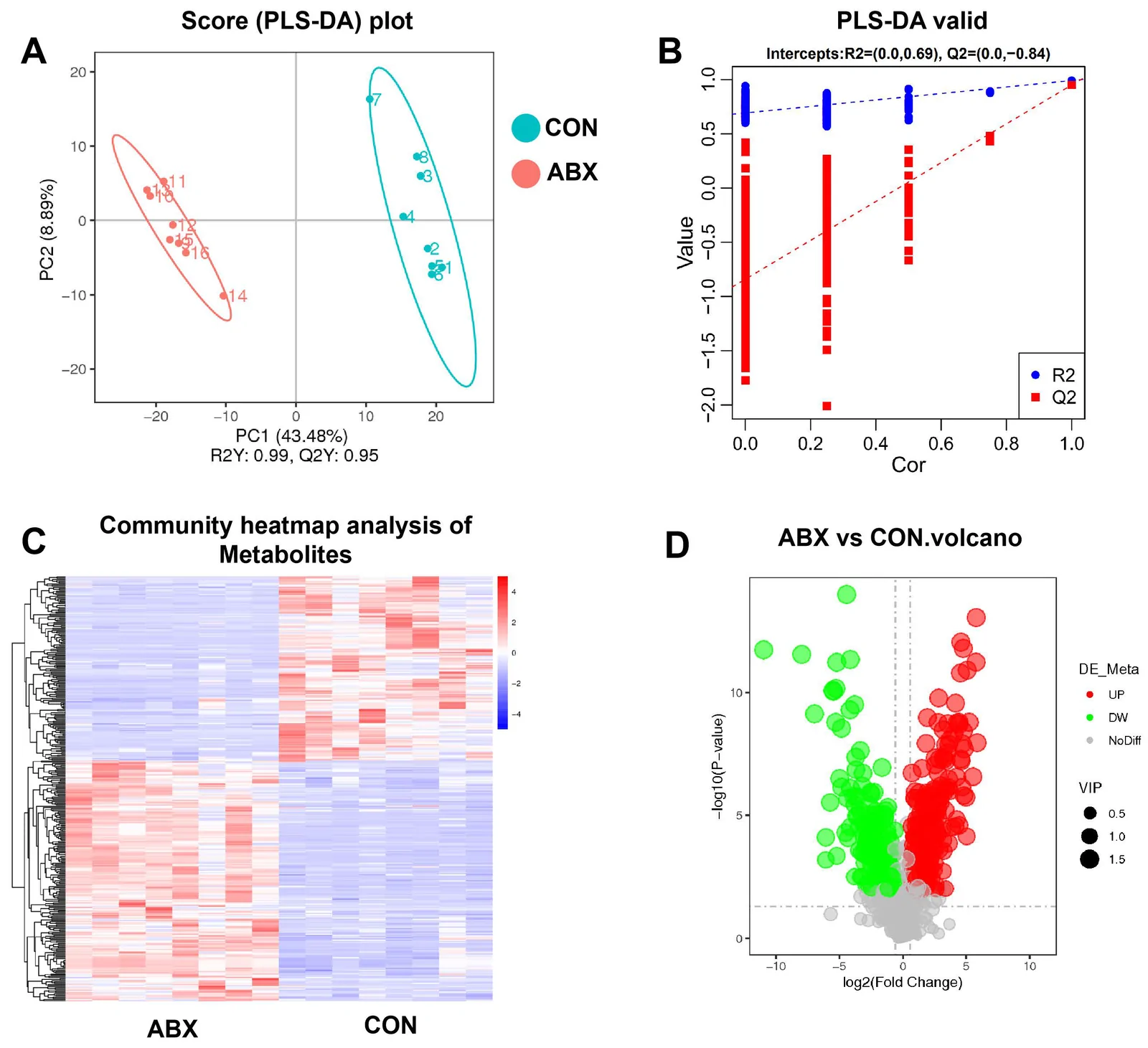
Overview of cecal metabolome changes induced by early-life ABX intervention (n = 8). (**A**) PLS-DA score plot of cecal metabolome; CON (cyan), ABX (red). (**B**) Permutation validation of PLS-DA model. (**C**) Heatmap of normalized metabolite abundance. (**D**) Volcano plot of differential metabolites between ABX and CON groups. Dot size represents VIP value. UP: significantly higher in ABX; DW: significantly lower in ABX; NoDiff: no significant difference.

Partial least-squares discriminant analysis (PLS-DA) was performed to characterize the global metabolic differences between the CON and ABX groups. The PLS-DA score plot shows clear separation of metabolic profiles between groups (Figure 6A). PC1 and PC2 explained 43.48% and 8.89% of total metabolic variation, respectively. Model parameters R^2^Y = 0.99 and Q^2^Y = 0.95 indicated the good explanatory and predictive power of the model. Permutation validation confirmed the reliability of the PLS-DA model, with intercepts of *R*^2^ = 0.69 and *Q*^2^ = −0.84 (Figure 6B). A metabolite-level community heatmap further illustrates the distinct metabolic signatures across samples (Figure 6C). Large-scale metabolic remodeling was observed, with numerous metabolites displaying group-specific abundance patterns between ABX and CON rats (Supplementary Table S1). A volcano plot was generated to visualize the differential metabolites (Figure 6D). Red dots represent significantly upregulated metabolites in the ABX group, green dots denote significantly downregulated metabolites, and gray dots indicate metabolites without significant changes. The dot size corresponds to the variable importance in projection (VIP) value. A substantial number of metabolites were differentially accumulated following early-life ABX intervention, demonstrating that the ABX intervention profoundly reshaped the cecal metabolome.

### 3.5. Characterization of Key Differential Cecal Metabolites upon Early-Life ABX Exposure

**Figure 7:**
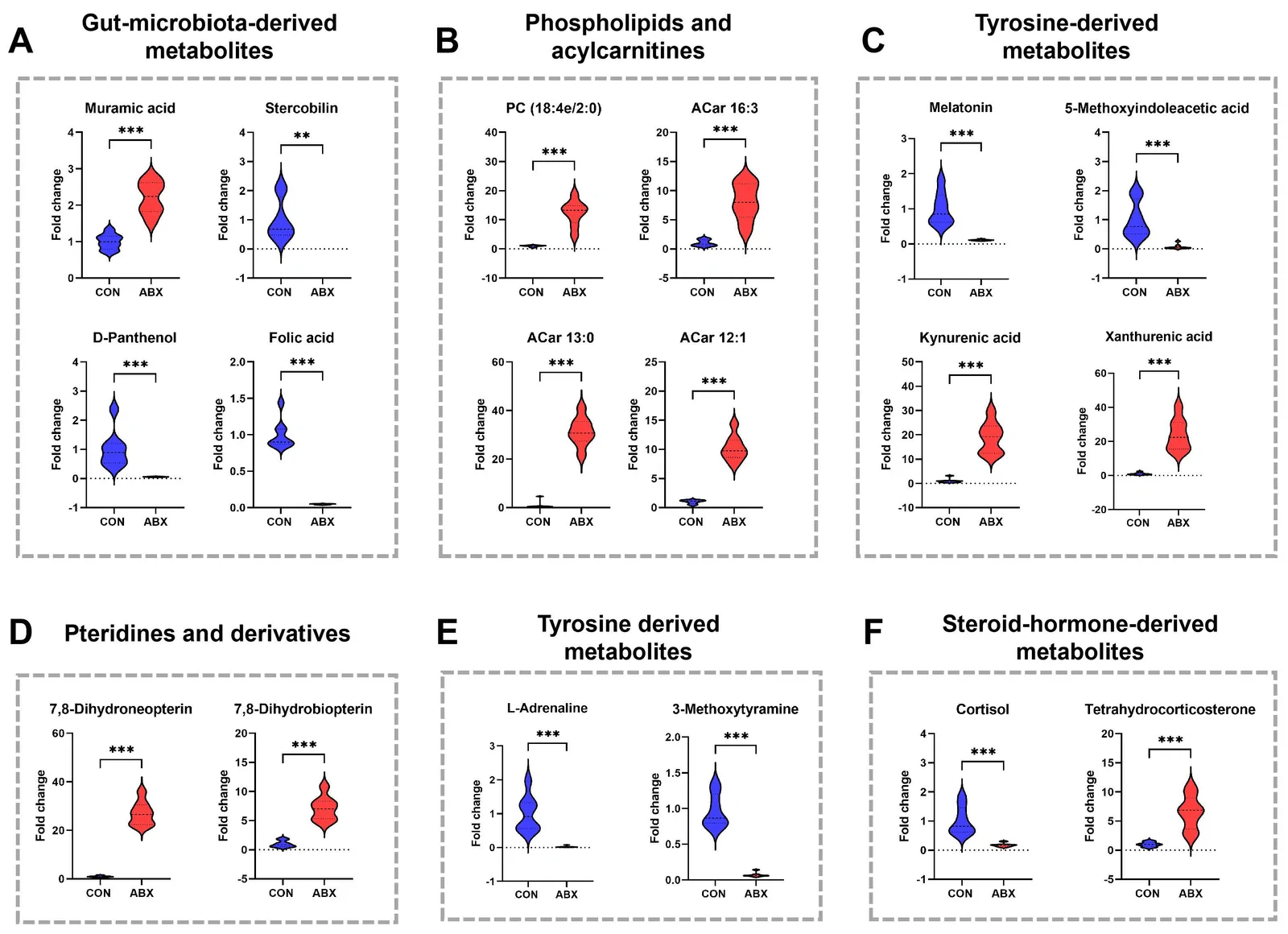
Violin plots showing fold-change of representative differential cecal metabolites grouped by metabolic pathway (n = 8). (**A**) Gut-microbiota-derived metabolites. (**B**) Phospholipids and acylcarnitines. (**C**) Tryptophan-derived metabolites. (**D**) Pteridines and derivatives. (**E**) Tyrosine-derived metabolites. (**F**) Steroid-hormone-derived metabolites. ** *p* < 0.01; *** *p* < 0.001.

Within gut-microbiota-derived metabolites (Figure 7A), muramic acid and stercobilin were significantly elevated in the ABX group. In contrast, D-panthenol and folic acid were markedly decreased after antibiotic intervention. The phospholipid and acylcarnitine classes (Figure 7B), including PC (18:4e/2:0), ACar 16:3, ACar 13:0, and ACar 12:1, all exhibited significant fold-changes in ABX-treated suckling rats. For tryptophan-derived metabolites (Figure 7C), melatonin and 5-methoxyindoleacetic acid (microbiota-related indole branch metabolites) were drastically reduced in the ABX group. Meanwhile, kynurenic acid and xanthurenic acid, two key metabolites of the host-dominated kynurenine pathway, were strongly upregulated under ABX treatment. Pteridine derivatives 7,8-dihydroneopterin and 7,8-dihydrobiopterin were significantly increased in the ABX group (Figure 7D). Among tyrosine-derived metabolites (Figure 7E), L-adrenaline and 3-methoxytyramine were significantly depleted following antibiotic exposure. For steroid-hormone-derived metabolites (Figure 7F), cortisol was downregulated, whereas tetrahydrocorticosterone was significantly accumulated in the ABX group. Collectively, early-life ABX intervention caused widespread perturbations of multiple metabolic pathways: depletion of microbiota-synthesized vitamins, indole-type tryptophan metabolites, and tyrosine-derived catecholamines; accumulation of acylcarnitines, pteridine intermediates, kynurenine pathway products, and partial steroid hormone metabolites. These pathway-specific metabolic shifts reflected profound disturbance of gut microbiota–host metabolic crosstalk.

## 4. Discussion

The early-life window of intestinal microbial colonization is pivotal for immune maturation, intestinal structural development, and lifelong metabolic programming [1,2,3]. To mechanistically dissect host–microbe interplay, antibiotic-mediated microbiota perturbation was utilized to establish antibiotic-perturbed dysbiosis models in suckling rodents. Consistent with prior reports, our present study further shows that the profound disruption of the indigenous gut microbiota during the lactational period substantially disturbs intestinal microecological homeostasis, alongside broad alterations in intestinal morphology, organ development, and cecal metabolic profiles [14,15]. Using a model with continuous oral-bolus antibiotic administration in neonatal suckling rat pups, we systematically characterized the microbiota-disrupting capacity of broad-spectrum antibiotic cocktails and identified microbial and metabolic shifts triggered by early-life microbiota perturbation. This work provides baseline phenotypic evidence to support the utility of lactational antibiotic-perturbed dysbiosis animal models.

Intestinal morphological and organ weight analyses revealed that early-life ABX intervention triggered mild structural impairment of the ileum, including blunted villi and expanded subepithelial spaces, whereas body weight and most visceral organ indices remained unaltered [17]. These observations highlight that the neonatal intestine is highly susceptible to microbiota perturbation, with immature intestinal architecture constituting one of the most readily detectable phenotypic responses to early-life microbial disturbance. Of note, the markedly elevated relative hindgut weight observed in the ABX group reflects the typical adaptive physiological compensation following microbiota deficiency, which aligns with the well-documented phenotypic features of antibiotic-perturbed and germ-free animals [18,19]. The preserved body and liver weights further indicate that short-term early-life antibiotic exposure does not produce overt systemic growth suppression or visceral organ injury. This implies that the intestinal microecological and metabolic changes detected in our study are predominantly driven by microbiota perturbation instead of non-specific antibiotic cytotoxic effects.

16S rRNA sequencing further confirmed that early-life ABX intervention severely impaired gut microbiota community assembly in suckling rat pups. The sharp reduction in OTU numbers, accompanied by significantly decreased α-diversity indices (Shannon, Simpson, Ace, and Chao), demonstrated that broad-spectrum antibiotic cocktail treatment significantly reduced microbial richness and evenness [20]. PCoA uncovered clear divergence in overall microbial community architecture between the CON and ABX groups, pointing to substantial microecological remodeling upon early-life microbial disturbance [21]. At the phylum level, Actinobacteria and Verrucomicrobia were markedly depleted following antibiotic exposure; these phyla encompass core beneficial commensals that support intestinal immune tolerance and microbe-derived vitamin biosynthesis [22,23]. At the genus level, multiple well-documented beneficial genera, including *Akkermansia*, *Bifidobacterium*, and *Lactobacillus*, alongside key members of the Ruminococcaceae family, were largely eradicated. In line with prior reports, *Akkermansia*, *Bifidobacterium*, and *Lactobacillus* constitute pivotal probiotic taxa that sustain intestinal microecological homeostasis [24,25]. Our findings verify that early-life ABX intervention substantially depletes these beneficial probiotic members, suggesting that the lactational antibiotic-perturbed dysbiosis model established herein is appropriate for follow-up mono-colonization assays and functional validation of key probiotic strains within this early-life developmental window. LEfSe analysis further identified the enriched pathobionts as core microbial biomarkers for antibiotic-triggered dysbiosis, whereas anaerobic beneficial commensals represented signature taxa of the immature, homeostatic gut microecology [26]. In addition, repeated oral-bolus handling, even using PBS vehicle, might trigger subtle stress-driven shifts in host metabolism and gut microbiota. Nevertheless, identical manipulation procedures were performed for both CON and ABX groups, so any handling-associated stress effects were balanced across experimental conditions and are unlikely to account for the observed inter-group differences. Taken together, these community shift profiles indicate that early-life antibiotic exposure elicits a characteristic dysbiotic phenotype marked by the depletion of beneficial anaerobic commensals and the expansion of opportunistic pathogens, which fundamentally disturb the ecological equilibrium within the developing intestine.

In parallel with this microbial dysbiosis, untargeted metabolomics further uncovered widespread metabolic disturbances within the cecum of suckling rat pups subjected to early-life microbiota depletion. ABX-driven microbial loss substantially lowered the abundance of microbiota-synthesized vitamins, including folic acid and D-panthenol. This observation underscores that the immature early-life intestine relies heavily on symbiotic commensal microbes to maintain vitamin metabolic homeostasis [23]. Furthermore, microbiota depletion strongly repressed the microbe-regulated indole branch of tryptophan metabolism, accompanied by diminished concentrations of melatonin and 5-methoxyindoleacetic acid. Meanwhile, the host-predominant kynurenine pathway became activated, resulting in the buildup of kynurenic acid and xanthurenic acid [16,27]. Such metabolic rewiring reroutes tryptophan flux away from immune-protective microbially derived indole metabolites toward pro-inflammatory host-synthesized kynurenine-related products. This metabolic reprogramming may partially explain the elevated susceptibility to immune-related disorders observed under conditions of early-life dysbiosis [27]. Apart from alterations in tryptophan metabolism, microbiota depletion also elicited broad-ranging disruptions of lipid metabolism, tyrosine metabolism, pteridine biosynthesis, and steroid-hormone metabolism. Pronounced accumulation of multiple acylcarnitines points to compromised intestinal lipid trafficking and energy homeostasis under microbiota-deficient conditions [28]. Increased levels of pteridine derivatives further hint at amplified intestinal oxidative stress and inflammatory tone following antibiotic exposure [28]. Additionally, depletion of tyrosine-derived catecholamines alongside perturbed steroid-hormone signatures demonstrates that early gut microbiota disturbance exerts far-reaching influences on host neuroendocrine and metabolic homeostasis [29]. When considered together with published data linking SCFA-producing bacteria to epigenetic programming in hematopoietic stem and progenitor cells, our metabolomic findings offer metabolite-scale mechanistic insights into how early microbial deficiency promotes long-term atopic immune phenotypes and developmental impairments [7]. When utilizing this antibiotic-perturbed dysbiosis model to probe gut microbiota–host metabolic crosstalk, researchers should bear in mind that antibiotic-driven microbiota depletion inherently induces intrinsic shifts in aromatic amino acid, lipid, and steroid metabolism. These confounding metabolic alterations ought to be carefully accounted for during experimental planning and result interpretation.

Compared with previous lactational antibiotic models, the present study systematically evaluated the microbial clearance efficiency and multidimensional metabolic characteristics of broad-spectrum antibiotics in suckling rat pups. Huang et al. reported that early microbial depletion regulates intestinal innate immune homeostasis through lipid-metabolite-mediated ILC3 differentiation, while our study further complements the overall microbial community variation and global metabolic disorder profiles, providing a more comprehensive landscape of early host microbe dysregulation [15]. Unlike conventional drinking-water antibiotic models suitable for weaned animals, the intragastric administration strategy adopted in this study precisely interferes with microbial colonization during the exclusive breastfeeding window, avoiding the interference of dietary factors and microbial adaptive recovery, which better simulates the pathological state of early clinical microbial dysbiosis [11]. Nevertheless, several limitations of this study should be acknowledged. First, although the antibiotic cocktail effectively perturbed most indigenous commensal bacteria and constructed a typical antibiotic-perturbed dysbiosis phenotype, residual drug-resistant bacteria still existed, which cannot completely mimic the sterile state of genuine germ-free animals. In addition, direct measurement of antibiotic concentrations in cecal contents or plasma was not performed in this study. Furthermore, serial intermediate sampling (e.g., PND 7) was absent, which prevented us from pinpointing the exact onset kinetics of gut phenotypic changes. Second, the current study only presents correlative relationships between microbial composition and metabolic phenotypes. Further culturomics-based strain isolation and mono-bacterial colonization or fecal microbiota transplantation verification are required to confirm the causal relationship between specific functional flora and host metabolic development. In addition, although pups were randomly assigned to treatments within each litter, statistical models were not built to explicitly adjust for litter-related variance. Potential litter-driven confounding originating from maternal background cannot be completely excluded. Further studies with an increased number of independent litters are warranted to better disentangle litter-specific influences versus treatment effects. Third, this study focuses on cecal microecological and metabolic alterations; future studies should further explore the distant regulatory effects of early intestinal dysbiosis on systemic immune development and multi-organ metabolic programming.

## 5. Conclusions

The present study demonstrates that early-life ABX-mediated microbiota depletion throughout the lactational period results in mild intestinal structural impairment, reduced microbial diversity, depletion of beneficial commensals, enrichment of pathobionts, and broad-ranging cecal metabolic dysregulation. Early-life microbial deficiency triggers multi-pathway metabolic reprogramming spanning vitamin, tryptophan, lipid, and hormone metabolism, consequently perturbing intestinal microecological and metabolic homeostasis. Collectively, these findings systematically delineate the microbial and metabolic phenotypic signatures of lactational antibiotic-perturbed dysbiosis models. They furnish robust experimental evidence for deciphering early-life host–microbe interaction mechanisms and deliver theoretical underpinnings for prudent clinical antibiotic stewardship as well as early-life microecological intervention strategies.

## Data Availability

The original contributions presented in this study are included in the article; further inquiries can be directed to the corresponding author. The 16S rRNA sequencing and metabolomics data were submitted to the National Genomics Data Center (https://ngdc.cncb.ac.cn/omix/preview/roqfLTGH; accessed on 10 August 2026) and are available in OMIX with the dataset identifier OMIX019457.

## Supplementary Materials

The following supporting information can be downloaded at: https://www.mdpi.com/article/10.3390/microorganisms14092048/s1, Table S1: The specific values of phylum-level relative abundance; Table S2: The specific values of the genus heatmap; Table S3: LEfSe scores between CON and ABX groups; Table S4: Detailed information on differential metabolites between CON and ABX groups. Figure S1. Stacked bar chart presenting relative abundance of gut microbiota at phylum level.
