# Supplementary material for: Early-Life Antibiotic Cocktail Intervention Alters Cecal Microbiota Composition and Metabolic Profiles in Suckling Rats
Source: Microorganisms. 2026 Sep 14;14(9):2048. doi: 10.3390/microorganisms14092048 (PMC13609107; doi:10.3390/microorganisms14092048)
Supplement: Supplementary file 1 [file microorganisms-14-02048-s001.zip › Supplementary Figure.pdf]

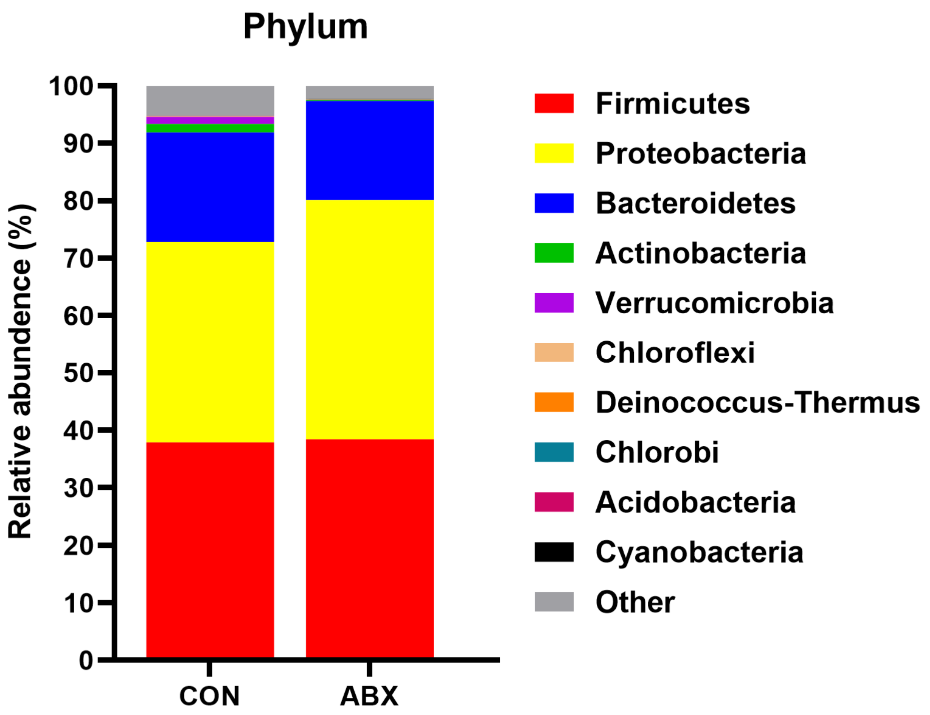

Figure S1. Stacked bar chart presenting relative abundance of gut microbiota at phylum level.
